# Bacterial response to graphene oxide and reduced graphene oxide integrated in agar plates

**DOI:** 10.1098/rsos.181083

**Published:** 2018-11-14

**Authors:** V. R. S. S. Mokkapati, Santosh Pandit, Jinho Kim, Anders Martensson, Martin Lovmar, Fredrik Westerlund, Ivan Mijakovic

**Affiliations:** 1 Division of Systems Biology, Department of Biology and Biological Engineering, Chalmers University of Technology, Kemivagen 10, Goteborg, Sweden; 2 Applied Chemistry, Polymer Technology, Chemistry and Chemical Engineering, Chalmers University of Technology, Kemivagen 10, Goteborg, Sweden; 3 Division of Chemical Biology, Department of Biology and Biological Engineering, Chalmers University of Technology, Kemivagen 10, Goteborg, Sweden; 4 WellSpect Healthcare, Aminogatan 1, Goteborg, Sweden

**Keywords:** agar plates, graphene oxide, reduced graphene oxide

## Abstract

There are contradictory reports in the literature regarding the anti-bacterial activity of graphene, graphene oxide (GO) and reduced graphene oxide (rGO). This controversy is mostly due to variations in key parameters of the reported experiments, like: type of substrate, form of graphene, number of layers, type of solvent and most importantly, type of bacteria. Here, we present experimental data related to bacterial response to GO and rGO integrated in solid agar-based nutrient plates—a standard set-up for bacterial growth that is widely used by microbiologists. *Bacillus subtilis* and *Pseudomonas aeruginosa* strains were used for testing bacterial growth. We observed that plate-integrated rGO showed strong anti-bacterial activity against both bacterial species. By contrast, plate-integrated GO was harmless to both bacteria. These results reinforce the notion that the response of bacteria depends critically on the type of graphene material used and can vary dramatically from one bacterial strain to another, depending on bacterial physiology.

## 1. Introduction

Different types of nanoparticles have been tested and used to study their anti-bacterial activity against various bacterial pathogens that are responsible for infections in humans [1,2]. After the discovery of graphene and its exceptional properties [3,4], it has also been reported that this material can act as an anti-microbial agent [5]. This has further resulted in synthesis and anti-bacterial testing of other derivatives of graphene such as graphene oxide (GO) with phenol, epoxide and hydroxyl groups on the basal plane [6] and reduced graphene oxide (rGO) [7], obtained by chemical treatment or by thermally annealing GO. Several studies on anti-bacterial activity of GO and rGO have been reported [8,9], where the mechanism of the anti-bacterial effect is attributed to the membrane stress induced by the sharp edges, resulting in physical damage to the bacterial cell [9]. Nevertheless, contradicting reports followed, some showing anti-bacterial activity of graphene derivatives [10] while some reporting the opposite [11]. According to the available literature, there are certain important parameters that decide the anti-bacterial activity of graphene, e.g. shape, surface functionalization, morphological state, number of layers, flake size, stability and properties of the underlying substrate [2,12–14]. Dose-dependent cytotoxicity on bacteria has been observed with many of the tested carbon materials [15–18]. Most studies on the anti-bacterial activity of carbon materials have been performed with GO because of its hydrophilicity and good dispersibility in water. Our aim in this study is to examine the bacterial behaviour on hydrophilic GO, and compare with the more hydrophobic rGO, in a solid-state set-up, where GO and rGO are integrated in agar plates, widely used by microbiologists. Therefore, we choose to work with two bacterial strains: *Pseudomonas aeruginosa*, which can reduce GO to rGO [19], and *Bacillus subtilis*, which is not capable of reducing GO to rGO [20]. In our experiments, GO and rGO were integrated in standard agar plates on which bacterial colonies were grown. Agar plates with rGO showed anti-bacterial effect against both *B. subtilis* and *P. aeruginosa*. By contrast, plate-integrated GO was harmless to both bacteria.

## 2. Experimental methods

### 2.1. Bacterial strain and culture medium

*Bacillus subtilis* NCIB 3610 and *Pseudomonas aeruginosa* PA01 were used in this study. Luria–Bertani (LB) broth/agar was used for the cultivation and growth for both strains. To prepare the inoculum, a single colony of each bacteria was inoculated in 5 ml of medium and incubated overnight at 37°C.

### 2.2. Graphene oxide and reduced graphene oxide

Single layer GO (dispersed in water) was acquired from Graphene Supermarket INC, USA. The concentration of the acquired GO is 500 mg l^−1^ with a composition of 79% carbon and 20% oxygen. The lateral flake size of GO is in the range of 0.3 to 0.7 µm. rGO was prepared in-house by using standard autoclave process. The so acquired GO was characterized using Raman (Alpha300 R) and FTIR (Perkin Elmer) analysis.

### 2.3. Preparation of agar plates with GO/rGO

LB agar plates were prepared using a standard protocol (10 g tryptone, 5 g yeast extract and 15 g of agar l^−1^). To prepare GO/rGO integrated plates, two sets of 0.01%, 0.02%, 0.04% and 0.08% of GO was prepared in sterile water and sonicated for 30 min. To reduce GO to rGO, one set of GO solution was autoclaved separately. Autoclaved LB agar medium was mixed with various concentrations of GO and rGO using a shaking incubator and poured into Petri dishes. Triplicates were used for all the experiments in this work.

Uniform dispersion of GO and rGO within the agar plates has been a constant concern. To overcome this, after the addition of GO and rGO to the agar medium, the solutions were kept under continuous stirring using a shaking incubator (20 min approximately), followed by pouring the solution in to plates for solidification.

### 2.4. Formation and analysis of colony biofilms

The overnight grown cultures of bacterial suspensions (2 µl) were inoculated on the agar plates containing various concentrations of GO and rGO and incubated at 37°C. The images of the biofilms were acquired after 1, 3 and 5 days of incubation and further processed with ImageJ 32 for the analysis of the total area of the biofilms. All experiments were performed in biological triplicates and presented as mean ± standard deviation.

### 2.5. Scanning electron microscopy analysis

Four-hour-old colonies grown with and without GO were collected and spread on a cover glass to make a thin film. The bacterial films were fixed with 3% of glutaraldehyde and dehydrated with graded ethanol as described previously [21] (electronic supplementary material). The dehydrated samples were then dried overnight at room temperature and coated with a thin layer of gold (5 nm) and observed under scanning electron microscope (JEOL JSM 6301F).

## 3. Results and discussion

### 3.1. Characterization of GO

**Figure 1.**
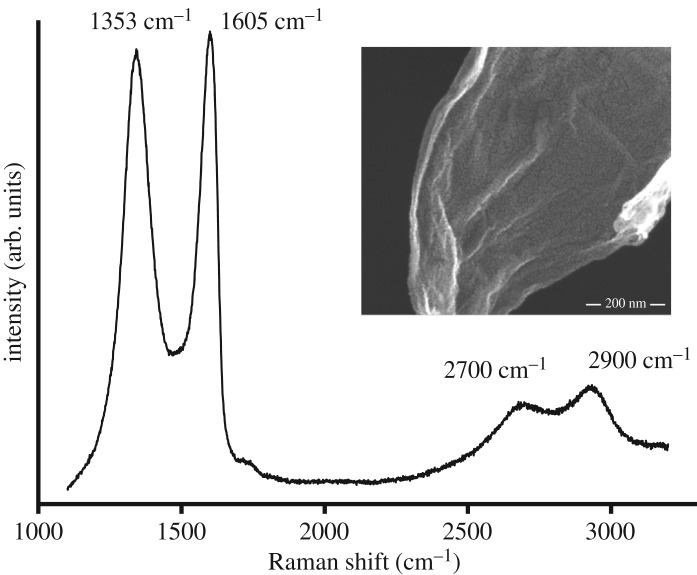
Raman characterization of acquired graphene oxide.

The acquired GO was characterized by Raman spectroscopy (figure 1). Typically, the G peak at 1605 cm^−1^ and D peak at 1353 cm^−1^ indicate the presence of GO. The peak at 1605 cm^−1^ corresponds to E_2 g_ phonon of SP^2^ carbon atoms, whereas the peak at 1353 cm^−1^ corresponds to k-point phonons of A_1 g_ symmetry [22]. In some cases, there could be a shift of G band and D band, which is an indication of certain defects, grain boundaries and other carbons. The ratio of these two bands and their intensity correspond to the quality of the respective graphene material. There are two other peaks observed at 2700 and 2900 cm^−1^, which corresponds to the graphene peak and second-order peak, respectively. If we observe the 2700 cm^−1^ band, it is slightly broad, indicating the presence of few-layer graphene (narrow peak indicates the presence of a mono- or bi-layer). The peak at 2900 cm^−1^ is derived from the combination of D–G peaks [22].

### 3.2. Detection of GO and rGO in agar plates

Agar plates with integrated GO and rGO were prepared as explained earlier. Presence of GO and rGO within the agar plates can be visually confirmed by dark precipitates of rGO (GO at higher concentrations also shows dark precipitates). These experiments used a rich medium void of glucose to facilitate rapid bacterial growth, since some studies state that glucose can reduce GO to rGO.

The agar plates with GO and rGO were characterized using ATR-FTIR to confirm the initial presence of GO and rGO, and the subsequent reduction of GO to rGO by the bacteria growing on the surface. To detect GO/rGO in agar plates before cultivating the bacteria, small samples of GO/rGO/LB agar were carefully cut using a lancet and mixed with KBr for ATR-FTIR analysis. After cultivating bacteria on the plates, the biofilm was carefully separated from the plate surface. Small samples of GO/rGO/LB agar from the surface that had been in contact with the biofilm were cut and subjected to ATR-FTIR analysis. (Keeping in mind the sensitivity of the samples, we have used the KBr method as it has a high transmission window and does not show any absorption spectrum in the IR region).

**Figure 2.**
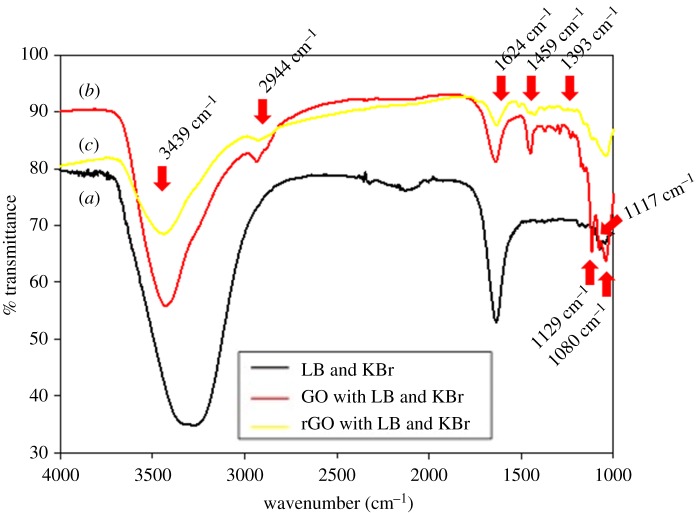
FTIR spectra of LB agar and KBr (black), GO with LB agar and KBr (red) and rGO with LB agar and KBr (yellow).

As seen in the transmission spectrum of figure 2, all the analysed samples show two strong peaks at 3439/3274 cm^−1^ (O–H stretching) and 1624 cm^−1^ (C = C aromatic ring) due to the adsorbed water and aromatic C = C. The characteristic peaks of LB agar and KBr along with O–H stretching at 3274 cm^−1^, C = C aromatic ring at 1624 cm^−1^ and C–N stretching at 1063 cm^−1^, were clearly visible in the FTIR spectrum of LB agar and KBr [23]. From the FTIR spectrum of GO sample, intensities of five characteristic peaks, at 2944, 1459, 1393, 1129 and 1117 cm^−1^, are higher compared to other samples. Several peaks are identical between GO and rGO: carboxyl C–O and O–H deformation was detected at 1393 and 1459 cm^−1^ in both samples.

For rGO, the intensities of the peaks associated with oxygen functional groups (at 1459, 1393, 1129 and 1117 cm^−1^) are found to decrease compared to GO because the functional groups of O–H deformation, carboxyl C–O and C–O stretching on the GO were destroyed by autoclaving or heating [24–26]. Additionally, in comparison to the FTIR spectrum of GO, the peaks at 2944 cm^−1^ (C–H or C–H_2_ stretching) and 1080 cm^−1^ (C–C stretching) of rGO almost disappear.

### 3.3. Toxicity of rGO towards *B. subtilis* and *P. aeruginosa*

**Figure 3.**
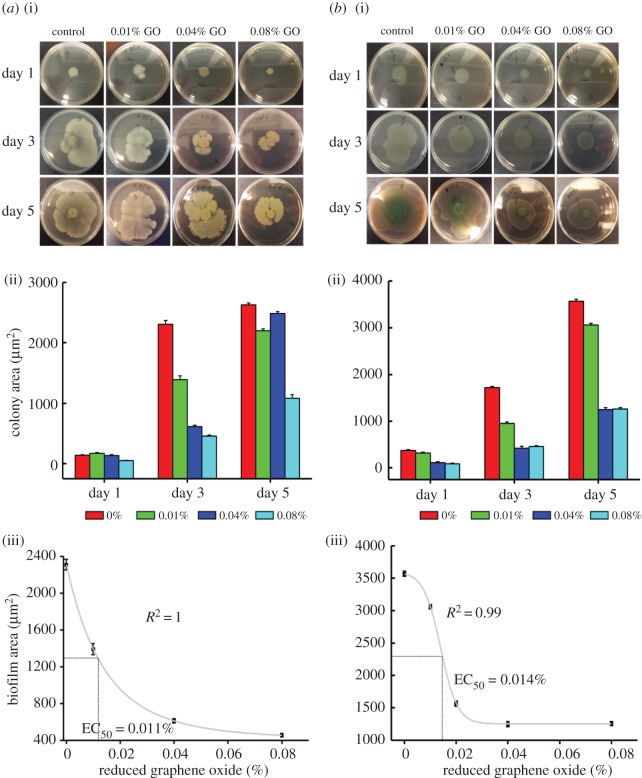
Photographs of *B. subtilis* (*a*) and *P. aeruginosa* (*b*) colonies cultivated on agar culture plates with different concentrations of rGO (0.01–0.08%) with seeded bacterial concentrations of 10^6^ CFU ml^−1^ (*a*(i),*b*(i)); measured bacterial biofilm area of each agar culture plate (*a*(ii),*b*(ii)). Sigmoidal plots of *B. subtilis* and *P. aeruginosa* with rGO. Inhibition of *B. subtilis* colony biofilm formation with increasing concentrations of rGO followed first order of exponential decay (*a*(iii),*b*(iii)).

Compared to other tested graphene materials, rGO has the highest oxidation capacity [10], which also attributes to its electronic properties. In our assays, rGO exhibited strong anti-bacterial activity against both *B. subtilis* and *P. aeruginosa* (figure 3). In our experiments, flake size would be expected to be irrelevant compared to the dispersibility and metallicity of the graphene material, which plays an important role when the flakes are in direct contact with cellular components [10]. Previously, it was observed that single-walled carbon nanotubes (SWNT) can mediate electron transfer over a lipid bi-layer [27] and this mechanism was experimentally proven by Liu *et al*. [10], showing that rGO can also form a conductive bridge and is capable of oxidizing cellular components.

The area occupied by the bacterial colonies was measured and correlated to the concentration of rGO (figure 3). The anti-bacterial effect of rGO on *B. subtilis* and *P. aeruginosa* is concentration dependent and the biofilm size of the treated samples was reduced drastically compared to the cells on control agar plates with increasing rGO concentrations. Correlation of rGO concentration versus *B. subtilis* colony area exhibited first order of exponential decay with correlation coefficient 1 (figure 3*a*). The correlation of rGO concentration to *P. aeruginosa* colony surface followed a sigmoidal pattern (*R*^2^ = 0.99) (figure 3*b*). The effective concentration of rGO to reduce the area of biofilm by 50% was 0.011% for *B. subtilis* and 0.014% for *P. aeruginosa*.

Another interesting observation is the presence of transparent white rings at the centre of the *P. aeruginosa* colonies. These rings contained mainly metabolically less active bacteria, and when these transparent zones were re-cultured in a non-graphene environment, they proliferated normally (electronic supplementary material, figure S1). From this observation, we presume that older, metabolically less active bacteria become more prone to killing by rGO. The glucose uptake from their environment [28] and the metabolic redox reactions [29–31] would be slowed down in these cells, making them less resistant to oxidative stress caused by rGO [10]. When the bacteria were separated and transferred to a graphene free environment, they could again divide and proliferate normally.

### 3.4. *B. subtilis* is resistant to GO, but *P. aeruginosa* appears to be susceptible

**Figure 4.**
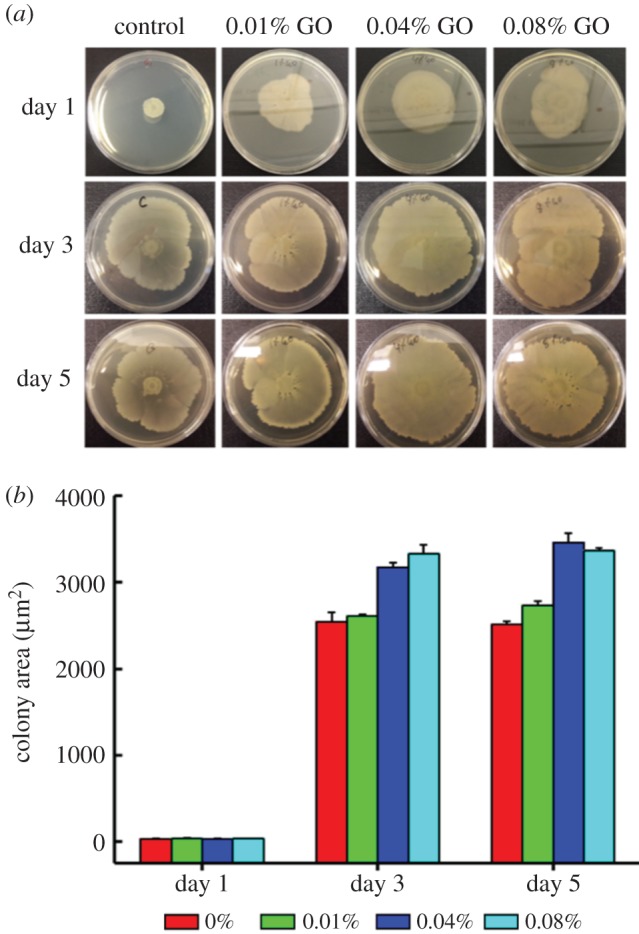
Photograph of *B. subtilis* colonies cultivated on agar culture plates with different concentrations of GO with seeded bacterial concentrations of 10^7^ CFU ml^−1^ (*a*); measured bacterial biofilm area of each agar culture plate (*b*). After day 3, the colony growth reaches a maximum threshold (saturation).

When grown on agar plates with integrated GO, *B. subtilis* was not adversely affected, and in fact its proliferation seems to be stimulated with increasing concentrations of GO (figure 4). This is in accord with previous reports stating that GO sheets can act as biocompatible sites for growth and proliferation of bacteria [32].

**Figure 5.**
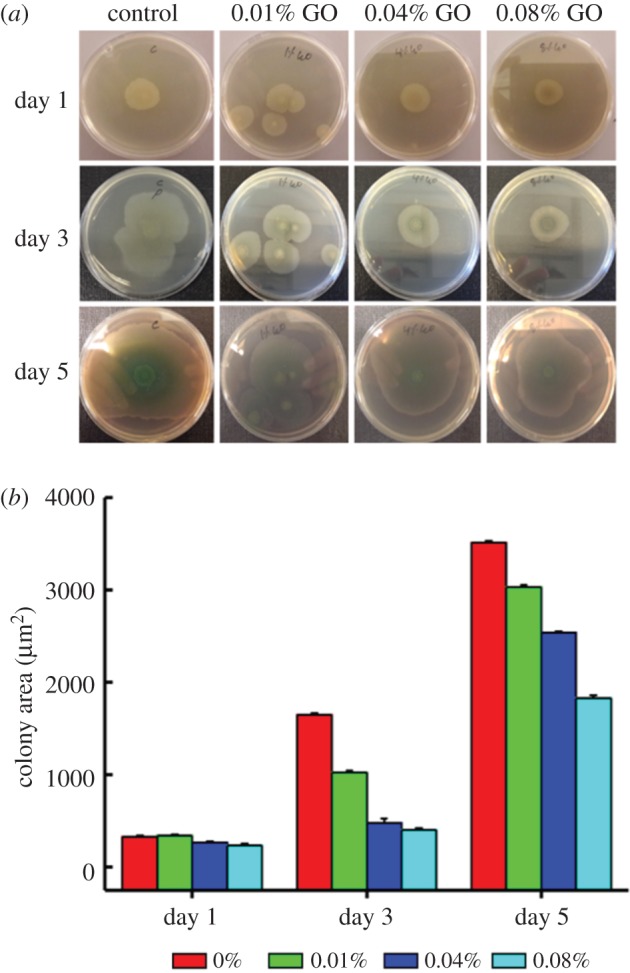
Photographs of *P. aeruginosa* colonies cultivated on agar culture plates with different concentrations of GO, the plates are seeded with bacterial concentrations of 10^7^ CFU ml^−1^ (*a*); measured bacterial biofilm area of each agar culture plate (*b*).

Next, we tested the effect of plate-integrated GO on *P. aeruginosa* (figure 5)*.* Initially, *P. aeruginosa* cells on GO integrated in agar plates started to proliferate and were unaffected for the first 24 h. Further, it was observed that bacterial growth became limited on plates with higher concentrations of GO. Presumably, during this time, *P. aeruginosa* engages in reducing GO to rGO as mentioned in the literature, without the help of any redox mediator [19]. Once enough rGO was produced, its anti-bacterial effect, as established in figure 3, became observable and the colony size was reduced with increasing concentrations of GO (figure 5; day 3 and day 5). At this stage (after day 3 and day 5), the supposedly rGO from the GO plates was isolated and subjected to ATR-FTIR. To our understanding, we could not detect any traces of rGO from either of the samples. This shows that either *P. aeruginosa* needs more time to reduce GO to rGO or there is not enough bacteria that could reduce GO to rGO in detectable quantities.

**Figure 6.**
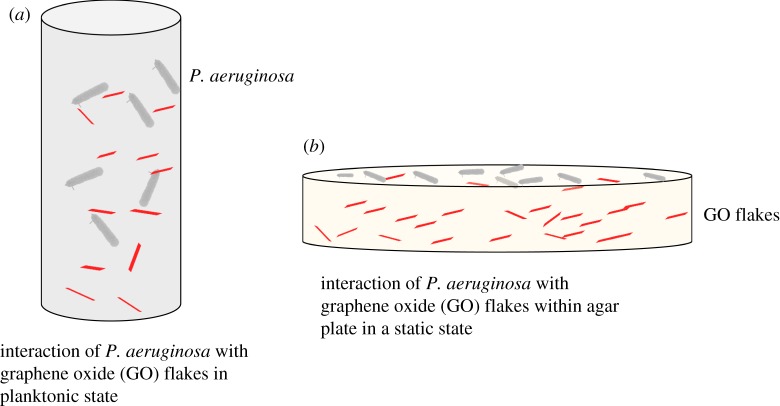
Diagrammatic representation of GO-bacterial interaction in planktonic (*a*) and static (*b*) environments.

Figure 6 shows the comparison of bacterial interaction with GO facilitating the reduction to rGO. In figure 6*a*, the bacteria are in the planktonic state and so the interaction with GO flakes is quite natural under continuous stirring [19]. Comparatively, in our case (figure 6*b*), the interaction of bacteria with GO is limited as they are in direct contact with only the GO flakes from the top layer. So, there are not enough bacteria that can convert GO to rGO in a static environment, unlike in the other case where they are under continuous stirring. We presume that this could be the main reason that we could not detect enough traces of rGO in the ATR-FTIR analysis.

**Figure 7.**
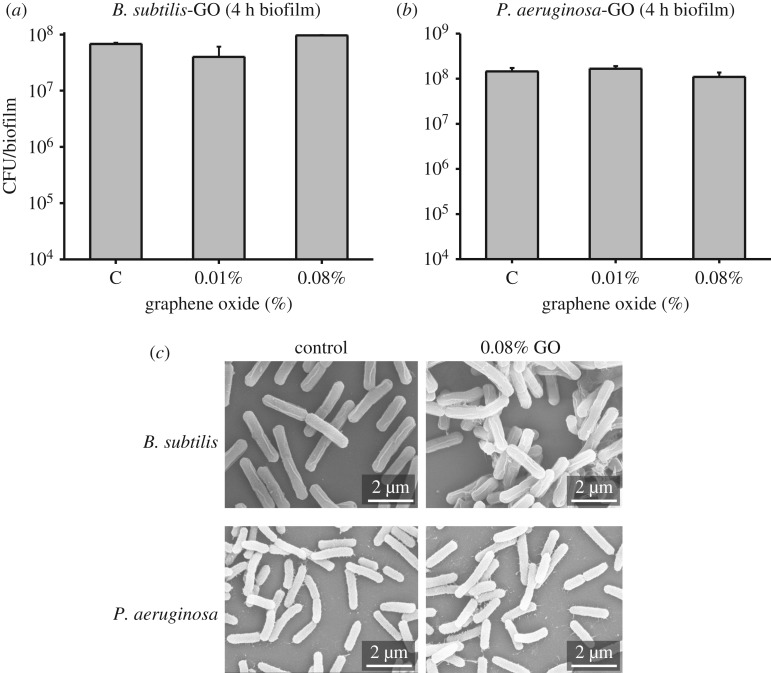
(*a*) *B. subtilis* and (*b*) *P. aeruginosa* biofilms were grown on LB agar (control) and (*c*) LB agar plates with integrated GO for 4 h. Biofilms were collected, homogenized and plated on fresh LB agar plates to count the colonies. For SEM, a small part of the 4 h biofilm was used. Imaging was performed after fixation, dehydration and drying, followed by deposition of 5 nm of gold.

According to our results, GO is completely harmless to *B. subtilis* and *P. aeruginosa* (until bacteria convert it to rGO). To confirm this, we examined the effect of GO on very early biofilm formation, after only 4 h. Four-hour-old colonies were homogenized with 0.89% of NaCl, diluted serially and plated on fresh agar plates to count the colony forming units. We did not observe any significant difference in the viability of *B. subtilis* and *P. aeruginosa* from plates with integrated GO compared to control (figure 7*a,b*). This supports the notion that the reduction of GO to rGO by *P. aeruginosa* does not start immediately, but rather accelerates with time and increasing number of bacteria. We also examined cell morphology in these samples by SEM, and there was no evidence of mechanical damage by GO to either *B. subtilis* or *P. aeruginosa* (figure 7*c*).

**Figure 8.**
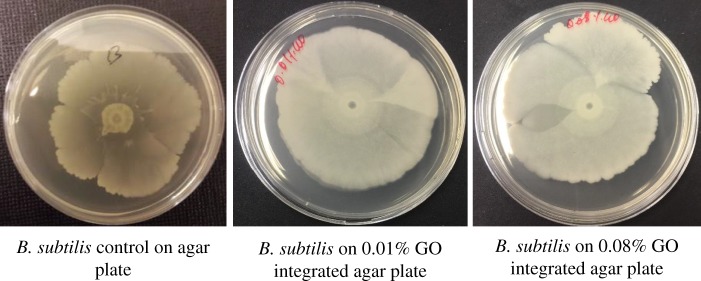
Proliferation of lysozyme-treated *B. subtilis* on agar plates with integrated GO. 2 µl of overnight grown *B. subtilis* was treated with 300 µg ml^−1^ of lysozyme and the suspension was inoculated on LB agar (*b*) and graphene oxide integrated agar plates and incubated at 37°C for 5 days.

Besides the above-mentioned possible capacity of *P. aeruginosa* to convert GO to rGO, there is another possibility to account for higher survival of *B. subtilis* on plates with integrated GO. The main difference between the cellular envelope of Gram-positive *B. subtilis* and Gram-negative *P. aeruginosa* is the thickness of their peptidoglycan layer. To assess whether the thick peptidoglycan confers any advantage to *B. subtilis*, we treated its inoculum with lysozyme, an enzyme known to degrade peptidoglycan. The lysozyme-treated *B. subtilis* was inoculated on agar plates with GO and cultured for 5 days and no significant difference in proliferation was observed between lysozyme-treated and -non-treated *B. subtilis* colonies on plates with GO (figure 8).

## 4. Conclusion

The anti-bacterial activity of GO and rGO against *B. subtilis* and *P. aeruginosa* was evaluated. GO and rGO were integrated with standard agar medium plates and characterized by ATR-FTIR. It was observed that rGO was toxic to both *B. subtilis* and *P. aeruginosa*, while GO was not harmful to either bacteria. However, growth of *P. aeruginosa* became inhibited on plates with GO after a certain time which could possibly be due to the reduction of GO to rGO by *P. aeruginosa.* When *P. aeruginosa*-treated GO was isolated and tested for ATR-FTIR, we could not detect any rGO which could be because of the fewer number of bacteria that could not convert whole GO to rGO in detectable quantities. This study highlights the importance in understanding the specifics of the interaction of graphene and its derivatives with bacteria, which can be purely mechanical but also metabolic. We hope that the methodology we have developed here, with GO and rGO flakes integrated in agar plates, will be useful for the toxicity assessment of other carbon materials and provide a basis for generating more comparable results across this field.

## Supplementary Material

Supplementary information

## Data accessibility

Most of the study is based on different imaging techniques and the measurement of the biofilm area. Relevant images are included in the manuscript and/or in the electronic supplementary material.
