# Supplementary material for: Bacterial response to graphene oxide and reduced graphene oxide integrated in agar plates
Source: R Soc Open Sci. 2018 Nov 14;5(11):181083. doi: 10.1098/rsos.181083 (PMC6281925; doi:10.1098/rsos.181083)
Supplement: Supplementary information [file rsos181083supp1.docx]

Bacterial response to graphene oxide and reduced graphene oxide integrated agar plates

V. R. S. S. Mokkapati^a^, Santosh Pandit^a^, Jinho Kim^a^, Anders Martensson^b^, Martin Lovmar^c^, Fredrik Westerlund^d^, Ivan Mijakovic^a^

**Supplementary figure**


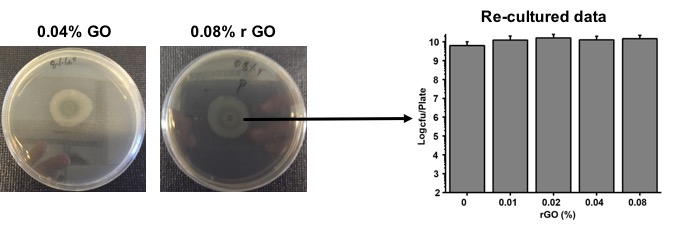


**Supplementary figure 1.** Photographs of *P. aeruginosa* colonies on GO and r GO which were taken for re-culture (left). Re-cultured data (right) which does not not show any significant difference on agar culture plates with different concentrations of r GO (right).

The center part of the 5 days colony biofilm of *P. aeruginosa* were removed carefully and homogenized on 0.89% of NaCl, diluted serially and plated on fresh agar plates to count colonies.

***Supplementary References***

Pandit S, Kim JE, Jung KH, Chang KW, Jeon JG: Effect of sodium fluoride on the virulence factors and composition of *Streptococcus mutans* biofilms. Arch Oral Biol 2011; 56: 643–649.
